# Spatial Indexing for Data Searching in Mobile Sensing Environments

**DOI:** 10.3390/s17061427

**Published:** 2017-06-18

**Authors:** Yuchao Zhou, Suparna De, Wei Wang, Klaus Moessner, Marimuthu S. Palaniswami

**Affiliations:** 1 Institute for Communication Systems (ICS), University of Surrey, Guildford GU2 7XH, UK; yuchao.zhou@surrey.ac.uk (Y.Z.); k.moessner@surrey.ac.uk (K.M.); 2 Department of Computer Science and Software Engineering, Xi’an Jiaotong-Liverpool University, Ren’ai Road Dushu Lake Higher Education Town SIP, Suzhou 215123, China; Wei.Wang03@xjtlu.edu.cn; 3 Department of Electrical and Electronic Engineering, University of Melbourne, Parkville, VIC 3010, Australia; palani@unimelb.edu.au

**Keywords:** mobile sensor data search, opportunistic sensing, mobile sensing, spatial indexing, Web of Things (WoT)

## Abstract

Data searching and retrieval is one of the fundamental functionalities in many Web of Things applications, which need to collect, process and analyze huge amounts of sensor stream data. The problem in fact has been well studied for data generated by sensors that are installed at fixed locations; however, challenges emerge along with the popularity of opportunistic sensing applications in which mobile sensors keep reporting observation and measurement data at variable intervals and changing geographical locations. To address these challenges, we develop the Geohash-Grid Tree, a spatial indexing technique specially designed for searching data integrated from heterogeneous sources in a mobile sensing environment. Results of the experiments on a real-world dataset collected from the SmartSantander smart city testbed show that the index structure allows efficient search based on spatial distance, range and time windows in a large time series database.

## 1. Introduction

The Web of Things (WoT) paradigm facilitates integration of the things and the data produced by them, paving the way towards context-aware solutions and smart cyber-physical systems. The growth in the number of connected things (a 2015 Ericsson report estimates 28 billion connected devices by 2021 [1]) will be accompanied with an explosive growth of data [2]. Since many applications can be translated to a set of data queries [3], the ability to retrieve valuable information from the mass of big data timely, efficiently and effectively is key to the success of cyber-physical systems. This translates to the need for efficient data retrieval services that can provide support for structured (or semi-structured) and rapidly changing content [2]. An important component of such data retrieval service is an elastic and scalable indexing system, as identified in a recent survey of WoT indexing models and techniques [2].

The emergent developments in domains such as smart cities, in which opportunistic sensing become more and more ubiquitous, bring new challenges for searching data in the WoT. Opportunistic sensing applications usually include mobile sources such as sensors mounted on city public transport and from citizens’ smartphones for participatory sensing [4]. As noted in a recent survey of WoT search techniques, the resulting data streams have dynamic properties that are “represented along the thematic, temporal and spatial dimensions” [5]. They are typically termed as ‘frequently updated, timestamped and structured’ (FUTS) data in the literature [4,6]. FUTS data cannot be treated as a pure time-series, as each time-stamped observation value may be associated with a different geo-location value, and is not obtained at successive, equally-spaced time points. FUTS data is usually defined in a structured format (e.g., JSON, XML or CSV), but the data models and schema adopted by the heterogeneous sources to describe the data are normally different and not always compatible. The resulting challenges for data search mechanisms can be summarized as follows: (1) data structures used for indexing must be compact to avoid excessive state exchange; (2) the indexing method must be able to deal with frequent updates of data from mobile sensing objects, and make it searchable immediately, while at the same time support “low-latency query evaluation” [2]; (3) queries should be able to request both current and historical data points for analysis; and (4) given the locality focus of WoT applications, proximity queries must be efficiently supported.

Existing works for querying sensor observation data usually search for the data source (sensor or smart objects) first and then establish communication channels to the selected objects to retrieve the data, as in SensorMap [7] Liveweb [8] GeoCENS [9]. Such methods are not efficient for many WoT applications that require complex data aggregation e.g., blending data from several sources. Moreover, they mainly focus on real-time monitoring, where only the most recent (and usually static) locations are recorded for data sources. For mobile data sources, retrieving historical data for previous locations may not be a simple process, or sometimes even not possible as the location information would have been over-written. Methods utilizing real-time indexing over data could be problematic for data with high update frequency or velocity, for example in incremental indexing, only modest update frequency is supported [2]. Another direction of research consists of approaches employing trajectory indexing to address the challenge of mobile objects in the WoT, which indexes the trajectories of moving objects [10,11,12,13]. However, the focus of trajectory indexing is the objects themselves, not the data generated by those objects. As such, these mechanisms do not address the requirements for updated locations with arbitrary timestamps, and cannot provide efficient time slice queries for aggregation.

To address the identified challenges, this paper presents a spatial indexing method for observation and measurement (O&M) data collected from mobile (as well as fixed) sensing objects. The method enables: (1) opportunistic sensing queries over historical and current O&M values. This facilitates data search in scenarios where there are no fixed installations of sensors in a geographical region. The data can be approximated by using mobile sensors that may have reported the needed data for the desired phenomenon (e.g., temperature) within a certain timeframe; and (2) time window-based queries over historical data with spatial constraints. The developed mechanisms have been evaluated on the SmartSantander (http://www.smartsantander.eu/) dataset, which is a real dataset with mobile sensing sources covering a large area of the city of Santander, in northern Spain. The experiments performed on this dataset show that the proposed method can successfully support both range and proximity queries effectively. Comparisons to the state-of-the-art methods in terms of standard evaluation metrics, i.e., index creation and query response time, reveal that it outperforms the existing methods.

The rest of the paper is organized as follows: Section 2 reviews and compares related work in sensor data search and supporting spatial indexing structures; Section 3 introduces the framework of the data search system and elaborates the design of the Geohash-Grid Tree and query processing; Section 4 presents the experiments and evaluation results in terms of index creation and query response time. The results are also compared to those produced with the state-of-the-art; Section 5 concludes the paper and outlines future research directions.

## 2. Related Work

A recent survey [2] of indexing mechanisms for the WoT identifies the R-family of data structures as suitable for spatial access methods. A resolution framework for IoT services based on the R-Tree data structure is presented in [14,15] where the operational areas covered by individual IoT services are mapped into indexing servers. A distributed hierarchical discovery architecture is outlined with catalogue servers storing the service areas of geographic indexing servers. On receipt of a discovery request, first the top-level catalogue server is queried which uses the specified geographic scope to select the set of indexing servers that have service areas overlapping the request scope. The request is then forwarded to the selected indexing servers with the rest of the service specification to be matched and the results integrated. As R-Tree-based indexing is unable to scale with rapid metadata updates, Wang et al. [16] overcome this limitation by indexing the gateways to which individual sensors connect (the gateways also implement semantic repositories storing the sensor descriptions) instead of indexing individual sensor service instances. Another semantics-based spatial search method [17] implements a distributed federated architecture of nodes, with each node encompassing local semantic storage, reasoning and search capabilities. The federated architecture is mapped to a hierarchical indoor location model, which allows scalable management of the large number of IoT devices. This approach, however, is tightly coupled to an indoor location environment, with semantic models describing logical locations and their relative positioning. The OSIRIS sensor Web discovery framework [18] proposes a combination of spatial, temporal (for temporal criteria in queries) and full-text (keyword-based search) indices to improve discovery performance. However, it is difficult to envisage how this approach can be applied in dynamic WoT environments which require frequent maintenance and update of the services. The above reviewed approaches are geared towards searching for WoT-enabled sensors or device descriptions, and not for the O&M values. Moreover, as stated in the WoT indexing survey [2], traditional database indices and distributed indices are deemed insufficient to deal with the WoT dynamics.

O&M data search is handled in SensorMap [7], Liveweb [8] and GeoCENS [9], which apply different indexing methods to the spatial information of sensors to support sensor search. The O&M data is then obtained by communicating directly to the selected sensors. O&M value queries are supported by the IoT-SVK [19] engine, which translates the sampled sensor observation values into keywords and then applies the value-Symbolized Keyword B+-tree indexing data structure to retrieve the O&M values. Queries for historical data are supported by indexing both the sensed O&M values and corresponding temporal information. The work in Linked Sensor Middleware (LSM) [20] focusses on sensor description and data annotations. Data provisioning, including historical data search, is supported through common interfaces. These O&M data search methods are not flexible to the dynamic WoT requirements as they assume that the O&M data context is static and not likely to change frequently.

Some works use prefix-overlapping hierarchical spatial (and temporal) structures to support spatial search for sensor data. The LOST-Tree [21] is a spatio-temporal data structure for managing sensor data loading and data caching on a client’s sensor web browser. It applies quadtree as the spatial framework and the Gregorian calendar as the temporal framework. A quadkey and a calendar string can be used to represent a spatio-temporal area. One of the notable features of the LOST-Tree is that it can avoid transmitting large amounts of sensor data repeatedly and therefore, make the sensor data loading process efficient. However, because of the highly dynamic nature of mobile sensing data and client queries, it is not clear how efficient the data loading process could be. The Aggregated Hierarchical Spatial (AHS) model [22] has been proposed to determine the topological relationships between sensor data and queries in publish/subscribe systems. With a predefined hierarchical indexing framework for (potentially complex) geometries, the AHS model can evaluate newly published sensor data against subscriptions in very efficient ways. However, it is reported that the indexing latency for point-based geometry is much smaller than other more complex geometry types, e.g., lines and polygons.

The research of trajectory indexing considers both spatial and time domain of moving objects, to enable multiple query functionalities. One research direction of trajectory indexing provides efficient query for the nearest trajectory to one or several points [10]. This approach stores approximated trajectories of moving objects and applies Discrete Wavelet Transform [23], and Chebyshev polynomials [24] to approximate trajectories better or index faster. However, the time series need to have the same length and the transform functions are not flexible for different queries [12].

Another research direction is to provide the most recent locations of moving objects. For example, the work in [11] provides a combination of spatial temporal R-Tree, hash table, and B-tree with MongoDB, aiming at enabling an efficient real time access to latest locations. The research direction that is similar to the approach proposed in this paper is indexing of historical spatio-temporal data. Two methods, Sampling and Update on change only, are mentioned in [13] to minimize update. However, the work for trajectory indexing always focuses on indexing objects, and does not provide a proper way of accessing data generated by moving objects. As a result, the design of trajectory indexing does not consider time slices and data aggregations functionalities, which makes answering the related query inefficient. Moreover, trajectory indexing sometimes just indexes proximity of moving objects’ trajectories. This is not suitable for analysis of data from opportunistic sensing as it loses information of the context of data.

### Comparison Table and Requirements for Mobile Sensing Environments

To provide a clear view on different methods for sensor data search and query in the literature, we summarize the characteristics of those methods, with a particular focus on mobile sensing data. Specifically, Table 1 explains the relevant metrics that are illustrative of the requirements, including data items, indexed domain, supported query, metadata update frequency, and query for historical data. The first three evaluate the search-related features of the corresponding indexing and query mechanisms, while the last two determine whether the reviewed methods provide support for mobile sensing environments.

Table 2 provides a comparison of the current state-of-the-art methods according to the metrics defined in Table 1. Based the study, we identify some additional challenges that need to be addressed by search mechanisms for mobile sensing data: (1) support for efficient metadata update of incoming data, especially spatial information produced by mobile sensing objects; (2) Support of query for historical data, which is important for data analysis such as detecting patterns and anomalies from sensing data in a particular geographical area over a particular time period; and (3) aggregation functionalities are vital for mobile sensing environments as data might not be measured in a synchronized way. In light of these requirements, we design a sensor data search framework which combines the Geohash-Grid Tree for indexing the spatial domain and Time-series Databases for control of the time domain, and provides query support with spatio-temporal constraints and aggregations on time-series data. The proposed method enables efficient indexing of spatial information from mobile sensing objects and supports different types of queries for historical data.

## 3. Data Retrieval Framework

FUTS data has a number of properties that can be used for searching: the phenomenon being measured (e.g., temperature, humidity etc.), the time and geographical location at which the measurement was recorded, the involved mobile sensing source (e.g., public bus), etc. The geographical property can be used to effectively reduce the search space, while others can be exploited to perform accurate search within the reduced search space. With this consideration, we build the data retrieval framework using geospatial indexing techniques and distributed O&M data repositories. We propose a spatial indexing mechanism as part of the data retrieval framework, particularly suitable for opportunistic and participatory sensing applications. Before presenting the data retrieval framework, we first introduce some of the important terms used throughout the paper and clarify their specific meanings in the context of this work.

**Definition 1** (Phenomenon). A physical property that can be observed and measured, whose value is estimated by applying some procedure in an observation (OGC O&M [25], SWE terms [26]).

Example: temperature being measured by a temperature sensor installed on the top of a bus to measure the temperature in the center of a city.

**Definition 2** (Virtual Object). Virtual Objects (VO) are virtualizations of Real World Objects (RWO) that have communication and interaction facilities with the surrounding environment and can provide real world data. A VO has metadata describing the associated RWO and O&M data indicating the status of RWOs or the environment.

Example: a virtualization of a public transport bus equipped with a temperature sensor to record temperature values along its route in a city.

### 3.1. System Overview

**Figure 1:**
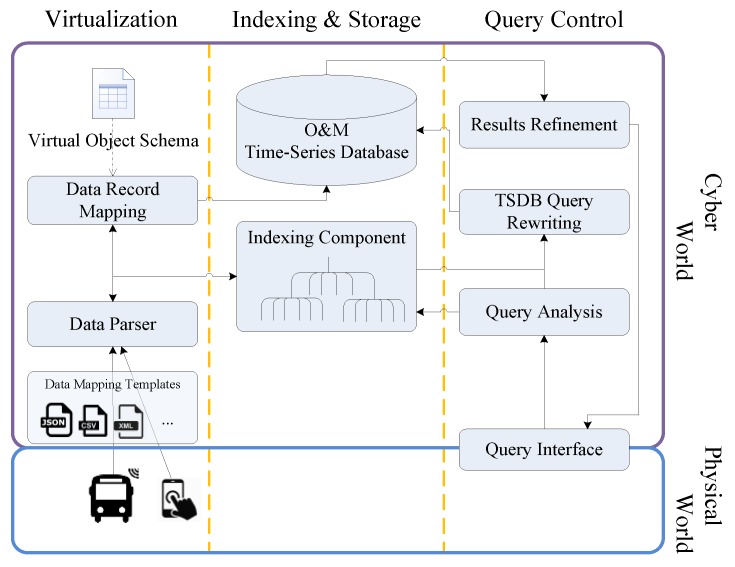
FUTS data retrieval framework.

Figure 1 shows a schematic representation of the functional blocks in the framework and their sub-components: (1) a virtualization component to control the data processing flow that parses the incoming data to extract the various features and feeds the extracted location values to the indexing component. It also maps the parsed data according to a virtual object schema, along with the geohash value generated by the Indexing component, to generate a data record to be stored in the time-series database; (2) an indexing component that enables efficient search based on spatial properties and returns the IDs of the selected VOs during query processing; (3) an Observation and Measurement (O&M) Time-Series Database (TSDB) to store the sensing data; (4) a query flow control component to analyse query features, distribute features to indexing component, rewrite the query to retrieve data from the TSDB, and refine the returned results from TSDB; (5) a query interface for interacting with users: specifying query parameters and presenting results.

**Figure 2:**
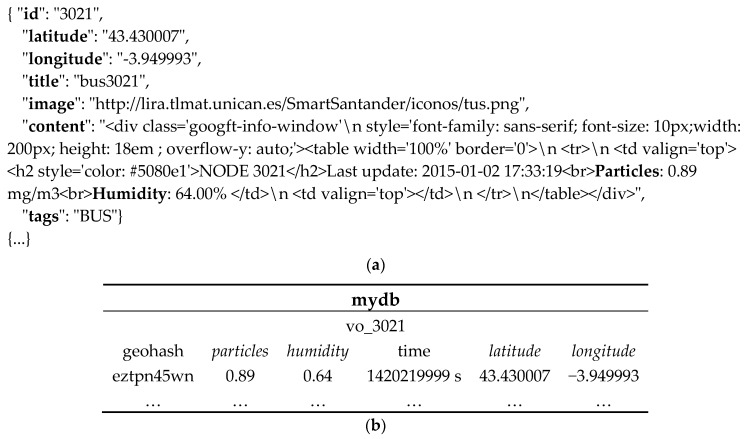
Example of Original Collected Data and its record in InfluxDB. (**a**) Example of Original Collected Data; (**b**) Example of Data Record in InfluxDB.

The framework supports FUTS data retrieval from a variety of data sources, which expose the data through Web interfaces. In our work, it collects data from mobile sensing objects in physical world (blue box), parses, processes, and manages the data in the cyber world (purple box). Since the schemas adopted by different sources may differ, a plug-and-play approach is adopted, with adaptors for extracting the data from heterogeneous sources. The adaptors utilize different scripts in various formats, e.g., JSON, CSV, XML etc., to parse the data. Our experience with sensor data streams (e.g., JSON data for SmartSantander and the Hypercat catalogue [27], CSV for London Air Quality Network [28] and a recent survey [29] on available sensor data streams for urban areas) shows the prevalence of these three representation formats. Figure 2 shows an example of the JSON data retrieved from the SmartSantander testbed and the resulting data record stored in the TSDB (the corresponding mapping template employs the gson (https://github.com/google/gson) deserialization library to convert JSON into Java objects).

To overcome the technological heterogeneity, the parsed data from fixed as well as mobile sensors is mapped to a virtual object (VO) representation. This allows applications to interact with the data without being concerned about the dynamicity of the underlying objects. Abstraction of sensing sources should differentiate between ‘data about things’ (i.e., things’ metadata, e.g., identity, location etc.) and ‘data generated by things’ (i.e., O&M data) [30]. The proposed VO model is lightweight and includes metadata related to the RWOs.

An abridged version of the VO model described in [4] is adopted in this work. Each VO is described with a unique identifier (ID) and a name. The associated O&M data is described by its associated phenomenon (e.g., temperature), actual measured value, unit of measurement, and time of the measurement. The measurement location information is captured in terms of a latitude-longitude pair and a geohash (http://geohash.org/) value. An example of VO instance is shown in Table 3.

In the following, we provide details on the two main parts of the framework: data repository mapping and query processing.

### 3.2. Data Repository Mapping

The data can be collected by using either available Web APIs or by engineering data transfer using URLs (cURL (https://curl.haxx.se/) scripts), and then parsed by the ‘Data Parser’ by applying relevant mapping JSON, CSV or XSL scripts. The location information from the parsed data, in terms of latitude-longitude pair, along with the VO-ID, forms the input to the Indexing component (described in Section 3.2.1).

The indexing component uses grids to partition the indexing space into equal-sized cells, which can achieve potentially good performance during updating and querying. It also generates the geohash value of the location information and returns it to the ‘Data Record Mapping’ component, which stores the generated geohash string, the O&M values and the associated metadata into the TSDB. The O&M data store is an InfluxDB (https://docs.influxdata.com/influxdb/v1.2/) time-series database that is optimized for high speed data ingest, query loads and data compression. It is available in both enterprise (Linux packages) and containerized (Docker image) versions. It offers write and query HTTP(S) APIs and a SQL-like query language for the aggregated data. To support FUTS data queries, which combine both spatial and temporal constraints, we extend InfluxDB with spatial query functionality.

As listed in Table 4, InfluxDB provides six concepts in its implementation. Databases in InfluxDB are independent of each other. To store data into or query InfluxDB, a specific database has to be chosen. Measurements are different groups of data, and are used for indicating the source virtual objects. InfluxDB is a schema-less data store, so measurements (comparable to tables in relational databases) do not need to have defined structures. Both tag and field (tag-key, tag-value, field-key and field-value) are columns in the table. Data in the same row in a measurement is called a data record. InfluxDB provides built-in indexing for tag-values (as string) and efficient query matching. Field-values are numeric and not indexed. Arithmetic comparisons of field-values are performed in a brute force way in the InfluxDB. The parsed VO information, including the measured environment phenomenon, their values and the associated timestamp, along with the generated geohash value, are reformatted into a data record before being written into InfluxDB.

**Definition 3** (Data Record). A data record is a 5-tuple of the form [<vo-id>, [<tag-key>=<tag-value>…(0..n)], [<field-key>=<field-value>…(1..n)], <geohash>, <unix-nano-timestamp>].

Thus, a data record consists of a vo-id, O&M measured values recorded in terms of at least one field-key and field-value pair, zero-to-many tag-key and tag-value pairs, the timestamp and the generated geohash. Figure 2a shows a snippet of an example FUTS data point and the resulting data record (Figure 2b) in InfluxDB.

#### 3.2.1. Geospatial Indexing for FUTS data from Mobile Sources

The core component of the proposed indexing method is the Geohash-Grid Tree structure, which is responsible for indexing spatial information of VOs. The tree stores the IDs of the VOs in leaf nodes whose spatial range covers the location of those VOs. The detailed information of VOs, formatted into data records, is stored in InfluxDB according to the data repository mapping mechanism, as described in the preceding section.

**Figure 3:**
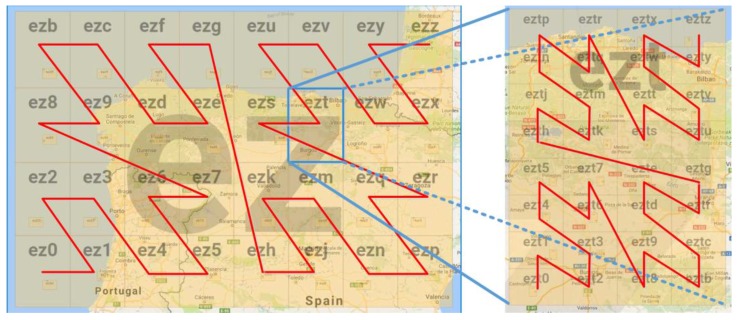
Z-order Filling Curve to Show Geohash Division. Maps are generated by using the Geohash Explorer service [31].

In the Geohash-Grid Tree structure, a node stores the prefix of geohash and the range it covers. Geohash is a geocoding algorithm that uses Base-32 (https://en.wikipedia.org/wiki/Base32) encoding and bit interleaving to convert latitude and longitude pairs to a string (which can also be reverted to the original latitude and longitude pair). A geohash is an encoded latitude and longitude pair representing a grid on the map. One bit divides the entire map area into two parts and adding more bits divides the map into more grids (e.g., 4, 8, 16, and 32). Since one character in a geohash string represents a 5-bit array, an area can have 32 grids in the order of Z as shown in Figure 3. For example, the grid of *ez* can be divided into 32 child grids, among which the grid of *ezt* can be divided into another 32 child grids. The longer the geohash string, the smaller the range of the grid and the higher the resolution. Geohash also represents a hierarchical structure, with a longer geohash string’s spatial range subsumed by a shorter geohash string with the same prefix. With different lengths of geohash, a geographical area can be divided into different number of grids with different sizes.

**Figure 4:**
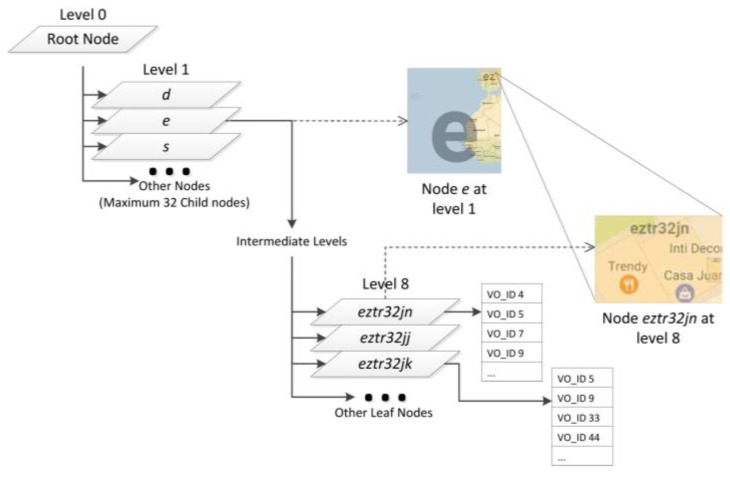
Geohash-Grid Tree Structure. Maps are generated by using the Geohash Explorer service [31].

The structure of the Geohash-Grid Tree is shown in Figure 4, in which a parallelogram represents a node of the tree. A node stores the two pairs of latitudes and longitudes of the corresponding map grid as well as its geohash. The rectangles (showing part of the maps) in Figure 4 indicate the geographical scope covered by the nodes. The maximum level of the tree is set as 8 and the root node is at level 0. Each non-leaf node of the tree can have at most 32 children and the leaf nodes are represented by 8-length geohashes. An example of the range covered by the leaf node is shown in Figure 4 with geohash “eztr32jn”, which covers around 200 m^2^ in Santander, Spain. A node in the Geohash-Grid tree specifies a fixed spatial range which covers the range of all its child nodes. There is no overlapping between nodes at the same level. The inserted IDs of the Virtual Objects (VO_IDs) are only stored in the leaf nodes.

Due to the mobile nature of the VOs, it is possible that the same VO_IDs may be stored at different leaf nodes, as shown in Figure 4 for VO_ID 5 and 9. In the worst case, when all the VOs have passed through all the map areas of a city and have reported observation data, every leaf node corresponding to the city area will store all the VO_IDs. In these extreme cases, the tree structure will not be able to provide efficient search based on spatial constraints. One possible solution to alleviate the problem is to make a new tree after some period so each leaf node of the tree is likely to store only a limited number of VO-IDs. The search process then knows which copy of the tree to search based on the temporal constraints in the query. This will impose additional storage requirement; however, it is not a challenging issue given the fact that the size of the tree is usually not large.

Algorithm 1 shows the algorithm for inserting a new value into the Geohash Grid Tree. During insertion, the tree receives a VO with a VO_ID and spatial information expressed as a latitude-longitude pair. The steps of indexing a VO into the tree structure can be summarized as follows:

*Step 1*: The VO_ID is inserted to the root node (Line 5). Upon receiving a VO, the node performs several checks: (1) the node checks whether the spatial information of the VO is contained in the range of the node (Line 7). If the VO is outside of the range of the node, the node ignores the insertion and the VO is discarded (Line 8); (2) the node is initialized if it is null (Line 10–12); and (3) if the node is a leaf node, VO_ID is added to the tree (Line 13–16).
*Step 2*: After Step 1, the location of the VO is confirmed to be within the range of the node. Then the node computes in which smaller range (the child node) the VO should be inserted. The smaller range, *subrange*, is computed by the node (Line 18).
*Step 3*: The node computes the *index* of a child node that matches the *subrange* (Line 20).
*Step 4*: At the end, the VO is inserted to the child node iteratively till the leaf node (Line 22). The node with the indexed VO will be returned (Line 13–16).

**Algorithm 1: Insertion in the Geohash-Grid Tree**INPUT: Virtual Object with VO_ID *vo_id*, latitude *lat*, and longitude *lon*OUTPUT: *rootnode* of tree*max_range* = *rootnode.getrange*();*geolevel* = 0;*rootnode* = *insert*(*rootnode*, *vo_id*, *max_range*, *lat*, *lon*, *geolevel*);**FUNCTION** *insert*(*node*, *vo_id*, *range*, *lat*, *lon*, *geolevel*) {  **IF** *lat* is outside of *range*.*latrange* || *lon* is outside of *range*.*lonrange*    **RETURN** *node*; // node unchanged  **END IF**  **IF** *node* == *null*    *node* = *createNode*(*range*, *geolevel*, *geohash*); // create new node in this level  **END IF**  **IF** *geolevel* == *maxlevel* // reach the maximum level    *node*.*entry*.*add*(*vo_id*);    **RETURN** *node*;  **END IF**  //Compute the smaller range that contains the location of VO  *subrange* = *computeRange*(*lat*, *lon*, *range*, *geolevel*)  //Get index of child_node that contains subrange  *index* = *computeIndex*(*subrange*, *range*)  // insert void to child_node, until reach the maxlevel  *node.getchild*(*index*) = *insert*(*node.getchild*(*index*), *subrange*, *vo_id*, ++*geolevel*);  **RETURN** *node*;}

A node only needs to be created when a VO, whose O&M location falls under the node’s spatial range, needs to be inserted. For example, assume a Geohash-Grid Tree has no nodes, if a VO reports a temperature value within the range of Node *eztr* (refer to Figure 3), the insertion will start from the root node and create Node *e*, Node *ez*, Node *ezt*, Node *eztr*, till a leaf node is created. The leaf node then creates a list to store the ID of the VO. The actual temperature value and associated metadata will be stored in InfluxDB. Subsequently, if another VO generates a temperature value within the range of Node *eztm*, just Node *eztm* and related nodes at the lower levels will be created, since nodes at upper levels already exist and do not need to be changed.

During insertion, the Geohash-Grid Tree either directly adds VO_ID to the matched list at an existing leaf node or creates a new leaf node to store the VO_ID. There is no need to update the existing tree structure to balance the tree, which accelerates the insertion process. In addition, since the VO_ID insertion is independent of the data records insertion in InfluxDB, the Geohash-Grid Tree also does not need to be updated every time when a VO (sensor) observation is generated, e.g., when the observation location of the VO is within the spatial range of the leaf node that already contains the ID of that VO. In this case, only a new data record is inserted into InfluxDB.

### 3.3. Query Processing

The query interface accepts a number of searching criteria, e.g., location of interest, observed phenomenon, temporal extent and aggregation functions for creating queries. The spatial filtering functions and temporal aggregation functions supported by the system include the following:

- Range queries—a rectangular area specified on a map, along with the desired time window.
- Distance queries—a circular area of interest specified as a point and a radius on the map within which observations are sought. Time window is also supported.
- Time window and aggregation—several temporal aggregation functions: minimum, maximum or mean of the stored O&M values within a given time window.

The details of the query processing procedure are shown in the algorithm in Algorithm 2. For data retrieval, the location information in the query is first used to search the spatial index to narrow down the potential VO-IDs. The query process first retrieves the matched nodes from the tree by checking whether the range of nodes is contained in or intersects with the range of the query (Line 4, Line 9–21). VO-IDs are retrieved from obtained nodes if there are any (Line 5, Line 23–33). This information, together with the environmental phenomenon, spatial range and temporal constraints, are converted into a template by the ‘Query Rewriting’ component, which serves as a pattern to query the relevant ‘measurement series’ in InfluxDB (Line 6). The actual O&M data retrieval based on the sliding time window is performed by InfluxDB (Line 7). The retrieved data records are parsed into a format appropriate for presentation in the query interface.

**Algorithm 2: Query Mechanism of Geohash-Grid Tree and InfluxDB**
INPUT: Query with env-phenomenon, *e*; range area, *r*; time window, *t*; a Geohash-Grid Tree, *tree*, with a *rootnode*.OUTPUT: *dataRecords* *listOfNodes* = *tree.getNodesbyrange*(*listOfNodes*, *rootnode*, *r*);*VO-IDs* = *tree.getVOsfromNodes*(*VO-IDs*, *listOfNodes*);*queryString* = *queryGeneration*(*VO-IDs*, *e*, *r*, *t*);*dataRecords* = *queryInfluxDB*(*queryString*); **FUNCTION** *getNodesbyrange*(*listOfNodes*, *node*, *r*){  **IF** *node.isempty*() or *node.exclude*(*r*)    **RETURN** *null*;  **END IF**  **IF** *node.containedIn*(*r*)    *listOfNodes.add*(*node*);  **END IF**// the node neither exclude nor contained in a range is intersect with the range  **FOR EACH** *child*_*node* of *node*    *child_range* = *child_node.getRange*();    *getNodesbyrange*(*listOfNodes*, *child_node*, *child_range*);  **END FOR**} **FUNCTION** *getVOsfromNodes*(*VO-IDs*, *listOfNodes*){  **FOR EACH** *node* in *listOfNodes*    **IF** node.level == maxlevel     *VO-IDs*.*add*(*node*.*getVOs*());    **ELSE**     **FOR EACH** *child*_*node* of *node*      *getVOsfromNodes*(*VOs*, *node.getChildNodes*());     **END FOR**    **END IF**  **END FOR**}


## 4. Experiments and Evaluation

In order to assess the effectiveness of the proposed spatial indexing mechanism, we evaluated it on a real-world, mobile sensing dataset collected from the SmartSantander smart city testbed. The results were also compared to state-of-the-art and the baseline geographical coordinates-based methods.

### 4.1. Dataset Description

The SmartSantander project provides a city-scale testbed for experimental research on smart cities. It offers various types of sensing data from both fixed and mobile sensors that support environmental monitoring of the city of Santander. Our experiments only made use of the data collected from mobile sensing devices installed on public transport systems, i.e., buses and taxis. These are 84 in total, corresponding to 84 VOs.

The dataset was created by scraping the map visualization of the sensor readings of the SmartSantander project (http://maps.smartsantander.eu/). The data was sampled 12 times each day (every two hours) for a period of 223 days, from 02 January 2015 to 13 August 2015. It consists of five different types of phenomena (environment qualities), i.e., CO, Humidity, Ozone + NO_2_, Particulate Matter (PM_10_), and Temperature. Each observation contains an ID of the mobile source and spatial and temporal information (i.e., where and when the observation was reported). There are in total 224,784 data records (corresponding to 1,123,920 observation values). However, since the data comes from mobile sensing objects, many issues arise such as loss of communications or battery power loss, which result in missing locations and observation values, and outliers in the collected data. Moreover, some VOs do not update measurements for some time periods. Therefore, the data was cleaned by removing all the records that have missing locations and observation values. Outliers were removed by setting thresholds for different sensor types. After cleaning, a complete dataset with around 100,000 records (corresponding to 500,000 observation values) was created.

The statistics of the dataset are illustrated in Table 5. There are in total 84 distinct mobile objects (buses or taxis attached with sensors) in the dataset, which are mapped to VOs, as described in Section 3.2. There are on average 448 data records per day, which are distributed almost uniformly. The locations of the data points are distributed within a 50-km distance in the city of Santander, with most of them clustered within the latitude range (43.4, 43.5) and longitude range (−3.9, −3.78).

For the experiments, 21 datasets were created, with the number of data records ranging from 1000 to 100,000, with increments of 5000. Different datasets have different time range; however, their location distributions are similar.

### 4.2. Experimental Settings

The proposed Geohash-Grid Tree approach was evaluated and compared to a widely used spatial indexing approach, R-Tree [32]. The Java Spatial Index (http://jsi.sourceforge.net/) library was used to implement the R-Tree index. It was also compared to the Geohash-based location tagging approach as detailed in our previous work [4] and the geo-coordinate location based approach using InfluxDB (as the baseline). In the geo-coordinate location based approach, the latitude and longitude values were part of the stored data records and were not indexed. The Geohash-based tagging method encoded the latitude and longitude as a geohash, and stored it as a tag column, which was indexed by the InfluxDB native indexing method. Experiments were carried out on different InfluxDB databases that stored different number of data records.

### 4.3. Evaluation Results

In this section, we present the evaluation results for the index creation and query processing. First the proposed Geohash-Grid Tree was compared to the R-Tree in terms of indexing creation time. Then the designed system was compared with the three other methods (detailed in Section 4.2) in terms of query response time.

#### 4.3.1. Index Creation Time

The first evaluation metric is the index creation time as it has a considerable impact on the performance of the data retrieval. The time taken for indexing using the Geohash-Grid Tree and the R-Tree was recorded for each datasets, with the number of data records varying from 1000 to 100,000. The tree structure was used to manage the spatial information of virtual objects. The indexing step also stored the VO_ID within each node. The experiments were repeated 20 times.

**Figure 5:**
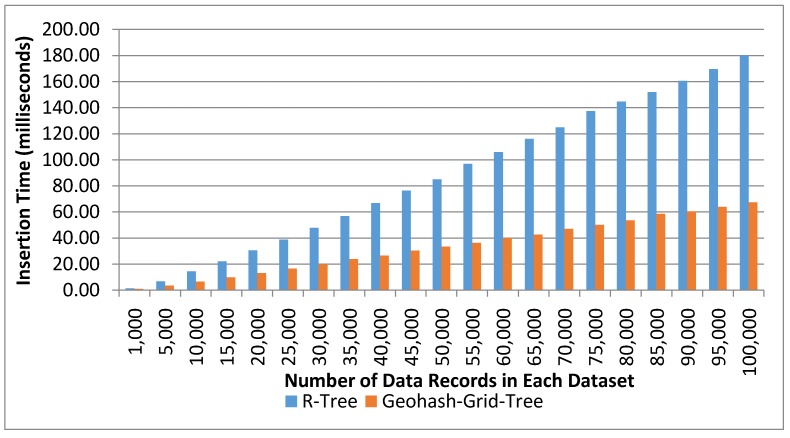
Index creation time: Geohash-Grid Tree and R-Tree.

Figure 5 shows the average indexing time using the two methods on different number of data records. Both tree structures took approximately linear time for indexing. R-Tree took around 180 ms to index 100,000 data records, while the proposed Geohash-Grid Tree method only took about 67 ms to index the same number of data records. The proposed method reduced around 43% time for indexing compared to R-Tree for 100,000 data records insertion. The index creation time is much shorter for the proposed method due to its fixed tree structure where indexed values are stored directly at the leaf nodes. It does not suffer from the tree balancing or reorganization problem, which is an expensive operation and inherent in the R-Tree.

#### 4.3.2. Tree Query Response Time

The second evaluation metric is the tree response time which measures the efficiency of data retrieval under certain spatial constraints. The response time used for retrieving the data records using the two methods was recorded for each dataset. Test queries were prepared for spatial point matching (i.e., find VOs at a specific point) and range matching (i.e., find VOs within a specific geographical area). The queries were tested in both sparse and dense areas; by ‘dense’ or ‘sparse’, we mean how dense or sparse the data records are at the specific geographical locations. Features of the test queries are summarized in Table 6.

**Figure 6:**
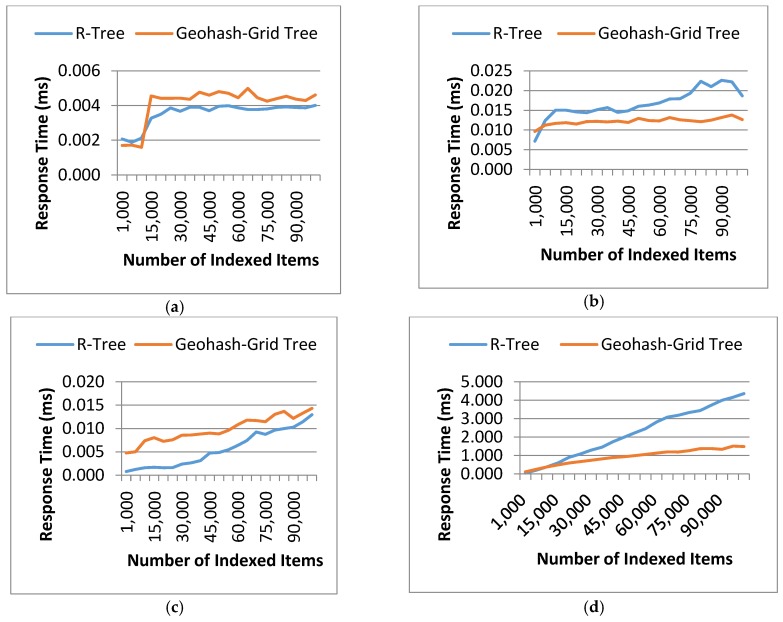
Tree response time: Geohash-Grid Tree and R-Tree. (**a**) Query 1: point matching in a sparse area; (**b**) Query 2: point matching in a dense area; (**c**) Query 3: range matching in a sparse area; (**d**) Query 4: range matching in a dense area.

The experiments were also repeated 20 times and the results were averaged and shown in Figure 6. In this experiment, four queries were carefully prepared: Query 1 and 2 to search at a particular point and Query 3 and 4 to search a large area. Query 1 and 3 were to search points in sparse areas, while Query 2 and 4 were to search in dense areas. As can be seen from Figure 6, query processing in Geohash-Grid Tree was more efficient in dense areas compared to the R-Tree structure (Figure 6b,d), while it was slightly worse in sparse areas (Figure 6a,c). This can be attributed to the fact that Geohash-Grid Tree uses a fixed height tree structure and there is no overlap between different nodes. On the contrary, ranges of nodes in the R-Tree overlap a lot in a dense area and little in a sparse area. Geohash encoding is only an approximation process and different levels of geohash have various precisions. The longer of the geohash string, the more precise of the encoding. The experiments applied a fixed length of 8 for the Geohash-Grid tree. This corresponds to errors in latitude of ±0.000085 degrees and longitude ±0.00017 degrees, i.e., around 40 m, which can be tolerated in city environmental monitoring applications. It is common that movement of the mobile sensors and objects is unpredictable and this often results in extremely unbalanced spatial distribution of the generated data. As the spatial distribution of data does not impact much on the Geohash-Grid Tree, it tends to be more scalable than R-Tree and thus more suitable for mobile sensing environments.

#### 4.3.3. Query Response Time

Query response time for the proposed data retrieval system using the Geohash-Grid Tree method was recorded and compared to the systems implemented with the R-Tree indexing, the Geohash_as_Tag and the Geo-coordinates_as_Field location retrieval based methods. All of the systems were implemented using InfluxDB as the data store.

Range queries were used to evaluate the query response time. A range query was specified by using an environmental phenomenon together with a spatial range area and a time window, i.e., query = {env-phenomenon, range area, time window}. The output was the observation and measurement values matching the query. The env-phenomenon is a type of measurement, e.g., Particles. The range area was represented by a rectangle with two pairs of latitude and longitude, which could be automatically retrieved from the map-based Query Interface of the framework. The time window was indicated by a start and end time.

Geo-coordinates_as_Field is a naive method that stores geo-coordinates as field in the InfluxDB, which considers fields as numeric values supporting arithmetic comparisons. Query matching can be done by directly comparing all the geo-coordinates of the data records with the constraints in the query.

Geohash_as_Tag is an approach to store spatial information by using geohash strings as tag-values. During retrieval, the Geohash_as_Tag method computes a geohash based on the spatial constraints of the query and retrieves data records with matching or overlapping geohash. For this method, the built-in InfluxDB functionality was used.

The query processing in the proposed data retrieval framework consisted of probing the Geohash-Grid Tree structure and InfluxDB sequentially, as described in Figure 6.

The steps involved in the R-Tree implementation were similar, with InfluxDB storing the actual latitude and longitude values of the data record as field. The generation of query string for InfluxDB in the cases of Geohash-Grid Tree and R-Tree used the same constraints as that for the Geo-coordinates_as_Field location retrieval method. One difference was that this query used the VO_IDs obtained from Geohash-Grid Tree and the R-Tree index, respectively, to retrieve answers, instead of using wildcards to query the entire InfluxDB database.

Table 7 shows the details of the test queries as well as the number of data records retrieved. It should be noted that these queries were prepared to test the performance of different methods in some extreme conditions, taking into consideration the characteristics of the datasets.

Query 1 and 2 set a short temporal range as the selection criteria, with 1-day and 5-day time window, respectively. Queries 3–5 set a longer time range, i.e., 6 months. Query 1 and 3 were specifically designed to search data in a relatively sparse area, and the number of returned data records was small. They were in the same spatial range, which contains just two indexed VOs in the largest dataset.

**Figure 7:**
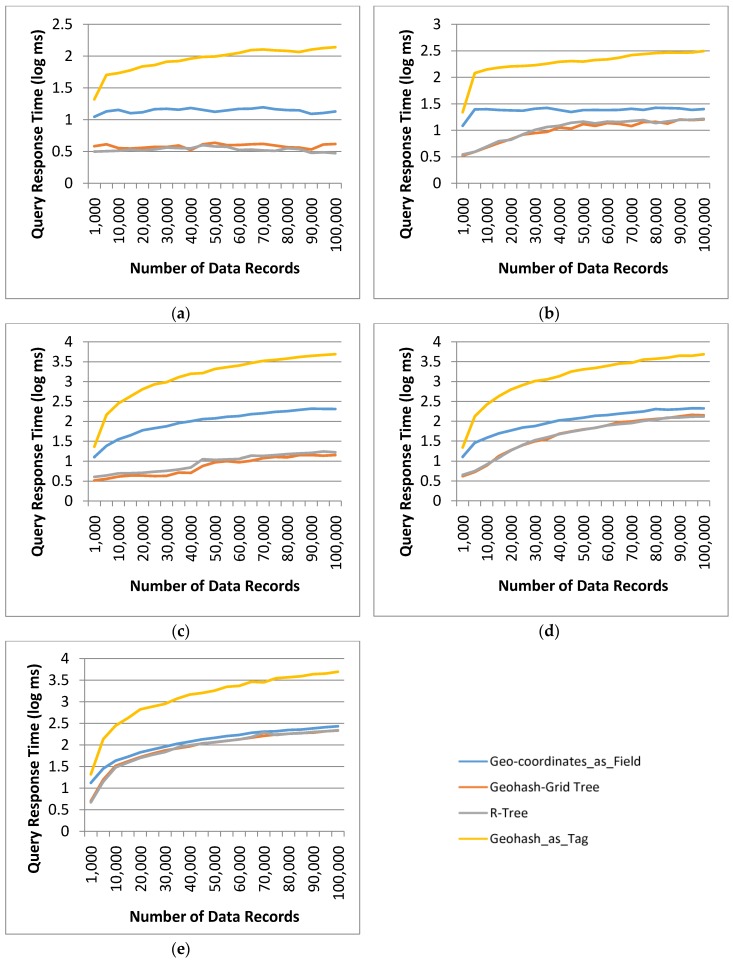
Query Response Time for different methods and selection criteria: (**a**) Query 1; (**b**) Query 2; (**c**) Query 3; (**d**) Query 4; (**e**) Query 5.

Query 1 returned 1 data record for 3 of the methods, this is because there is just one data record for the temporal parameter specified in the query but all the VOs in the dataset fall within the spatial range of the query (hence, are returned by the indexing tree structure). Queries 2 and 4 retrieved data in a relatively dense area and retrieved 46 VOs. Query 5 specified a long temporal range (6 months) and a spatial range in a dense area, and retrieved 58 VOs. As the VOs in the spatial range of Query 5 repeatedly traversed the area, it retrieved much more matched data records than Query 4. The results for the query response time using the four different methods were plotted in a log scale in Figure 7. The indexed Geohash_as_Tag approach performed the worst, taking 138.30 ms for Query 1, 313.37 ms for Query 2, 4895.08 ms for Query 3, 4852.11 ms for Query 4 and 4972.48 ms for Query 5 for database containing 100,000 data records. These numbers were an order of magnitude different from that of the other methods. The large response time was due to the fact that generation of an overlapped geohash string enlarged the spatial range significantly. This resulted in many non-related data records that were outside the spatial range of the query, being retrieved from InfluxDB. This can be seen from Table 7, for Query 1, 616 data records were retrieved from the dataset, and for Query 5, 93,404 data records were retrieved from the largest database in the experiments.

All the other three methods returned the same number of data records for all queries (the numbers of the largest test dataset are listed in Table 7). Since the dominant cost of the query response time was the query time in InfluxDB, the performance difference between the two methods using Geohash-Grid Tree and R-Tree couldn’t be seen clearly. From Figure 7, it also can be seen that if the number of VOs retrieved from tree indices was large (close to the total number of VOs in the dataset), the query response time of the proposed method tended to close to that of Geo-coordinates_as_Field based method, for example, in Query 5, 58 VOs were retrieved and there were only 84 VOs in the dataset.

## 5. Conclusions

The presented work provides a fundamental solution to sensor data search and query, which is important for many WoT applications; especially those that employ opportunistic and participatory sensing for data analysis purposes. For example, for environment monitoring in smart city applications, a large amount of opportunistic sensing data can be collected, stored, processed and analysed to discover useful knowledge both in (near) real time and long term. Based on this, we could derive pollutant concentrations everywhere in the city at all times and present the implications for health risk to everyone. As a key component of the mobile sensing data search framework, the proposed Geohash-Grid Tree uses grids to partition the indexing space into equal-sized cells, which enables efficient index update and query. The data search framework is particularly suitable for opportunistic sensing queries over historical and current O&M values with spatial constraints. The experiments and evaluations showed that the Geohash-Grid Tree method is scalable, and the proposed method outperforms the existing approaches in terms of index creation and retrieval of a substantive number of data records over a large span of time. For shorter time periods, the performance of the proposed approach is comparable to the R-Tree based indexing.

Processing and analyzing mobile sensing data is a challenging and complex problem and data search is one of the first steps in dealing with such a problem. One important objective of our future research is to extract patterns and anomalies from both mobile and fixed sensing data collected from a city’s environment. Usefulness of the mobile sensing data has already been shown in some research works, such as analyzing traffic anomalies in cities by integrating mobile sensing data and social media data [33], and monitoring air quality urban areas [34]. Our work aims to design methods for trustworthy knowledge discovery by harmonizing data and information collected from not only the physical, but also the social and cyber worlds. Our current implementation of the Geohash-Grid Tree only focuses on point-based sensor observations. However, sensor observations and queries can also be specified by using more complex geometries, such as lines or polygons. There has been research on developing hierarchical spatial data structures and topological operators in deriving relationships between complex geometries in publication/subscription settings [22]. In our future work, we plan to extend our current design to support queries with more complex spatial constraints and to design more suitable representation and visualization methods for the results of those queries.

## Figures and Tables

**Table 1:** Metrics of Indexing and Query.

Metrics	Descriptions
Data Items	Indicates the items managed by the reviewed system. They are the objects used for the indexed domains and corresponding query functionalities.
Indexed Domain	Spatial, temporal, or thematic domains indexed by the system.
Supported Query	Query functionalities supported by the system.
Metadata Update Frequency	Frequency of update for the metadata of sensor data, especially spatial information. Frequently changing spatial information is one of the key characteristics in mobile sensing environments and a good system should support high metadata update frequency.
Query for Historical Data	Whether the reviewed system supports queries for historical data. Historical data is important for data analysis.

**Table 2:** Comparison Table of Related Work.

Search Method	Data Items	Indexed Domain	Supported Query	Metadata Update Frequency	Query for Historical Data
IoT service resolution framework [14,15] (R-tree)	IoT Service metadata	Spatial	Point or area-based spatial query	Slow	-
Wei et al. [16] (R-tree)	Sensor metadata	Spatial	Point-based spatial query	Very fast	Not supported
OSIRIS [18]	Sensor metadata	A spatial Index for spatial domain, a temporal index for temporal domain, a full-text index for thematic domain	Sensor instance discovery and sensor service discovery based on search criteria of metadata, including spatial, temporal, and thematic metadata	Medium	Yes
SensorMap [7]	Sensor metadata	Spatial	Spatial search for latest values generated by sensors	Medium	No
Liveweb [8]	Sensor data	Keywords indexing for thematic domain, binary search tree for values	Search for real-time content based on keywords, category, and value range	Slow	Yes
GeoCENS [9]	Sensor Web Service	Spatial filling curve for spatial domain	Geospatial search based on key-value pair queries	Slow	Yes
IoT-SVK [19]	Sensors, devices, objects	Spatial-Temporal R-Tree for spatial and temporal domain, B+-Tree for keywords and values	Keyword-based search Spatial-temporal search Value-based search	Fast	Yes
Linked Stream Middleware (LSM) [20]	Sensor streams as RDF triples	-	SPARQL-based continuous query with semantic constraints (including spatial and temporal domain constraints)	Slow	Yes
LOST-Tree [21]	Sensor data and geometries	Spatial, Temporal	Spatial and temporal queries	-	-
AHS model [22]	Sensor observation data	Spatial	Spatial query with complex geometry	Very fast	Not supported
Bouros et al. [10]	Trajectories	Spatial and temporal	Retrieval of the top-k trajectories that pass as close as possible to all query points.	Fast	Yes
Chan et al. [23]	Haar Wavelets transformed trajectories	Spatial and temporal	Range query and nearest neighbour query for trajectories	Fast	Yes
Cai and Ng [24]	Chebyshev approximation of trajectories	Spatial and temporal	K nearest neighbours query for trajectories	Fast	Yes
Chen et al. [12]	Trajectories	Spatial and temporal	K nearest neighbours query for trajectories	Fast	Yes
Zhu and Gong [11]	Trajectories	Spatio-temporal R-tree for spatial and time domain,	real-time access to latest trajectories, trajectory-based queries	Fast	Yes
Proposed Approach	Virtualized objects and sensor data	Geohash-Grid Tree for spatial domain, TSDB for time domain	Query with spatio-temporal ranges and phenomenon, Aggregations on time-series data	Very fast	Yes

Note: “-“ means the feature is unknown or not made explicit from the paper.

**Table 3:** Example of VO instance.

**ID**	3021	**Name**	bus3021
**Type**	http://dbpedia.org/page/Bus	**Mobile**	Yes
**Location {Latitude, Longitude, Geohash}**			
{43.430007, −3.949993, eztpn45wn}			
**Information {Name, Value, Unit of Measurement, Time, Description}**			
{Particles, 0.89, mg/m^3^, 02 January 2015 17:33:19, density of particles with a diameter between 2.5 and 10 micrometres}			
{Humidity, 0.64, percentage, 02 January 2015 17:33:19, Humidity}			

**Table 4:** Storage Mechanism of InfluxDB.

Database	Measurement	Tag-key	Tag-value	Field-key	Field-value
mydb	vo_3021	geohash	eztpn45wn	*humidity*	0.64

**Table 5:** Statistics of the collected SmartSantander Data.

Dimension	Total	From	To	Distribution
VO	84			
Environmental Phenomenon	5			CO, Humidity, Ozone + NO_2_, Particles (PM10), and Temperature
Latitude	827	42.9855	43.6636	Largely distributed between 43.4 to 43.5
Longitude	9978	−4.13309	−3.53594	Largely distributed between −3.9 to −3.78
Date	223 distinct days	Friday, 02 January 2015 17:33:19 GMT	Thursday, 13 August 2015 11:59:09 GMT	Almost a uniform distribution

**Table 6:** Query Constraints and Number of Query Results from Tree Structures.

Query constraint	Query 1	Query 2	Query 3	Query 4
Point	(43.1702, −3.89954)	(43.4632, −3.80883)		
Location range			(43.4, −3.6) to (43.5, −3.5)	(43.4, −3.9) to (43.5, −3.8)
Indexed items density in nearby area	sparse	dense	sparse	dense
Number of distinct VO_IDs of returned indexed items	1	1	1	84

**Table 7:** Query Constraints and Number of Query Results.

Phenomenon	Query 1	Query 2	Query 3	Query 4	Query 5
	Temperature				
Time_from	21 January 2015 10:00 a.m.	21 January 2015 10:00 a.m.	21 January 2015 10:00 a.m.	21 January 2015 10:00 a.m.	21 January 2015 10:00 a.m.
Time_to	22 January 2015 10:00 a.m.	26 January 2015 10:00 a.m.	22 July 2015 10:00 a.m.	26 July 2015 10:00 a.m.	22 July 2015 10:00 a.m.
Time range	1 day	5 days	~6 months	~6 months	~6 months
Location range	(43.4, −3.94) to (43.42, −3.93)	(43.47, −3.79) to (43.473, −3.785)	(43.4, −3.94) to (43.42, −3.93)	(43.47, −3.79) to (43.473, −3.785)	(43.467, −3.79) to (43.47, −3.787)
Data records density of nearby area	sparse	dense	sparse	dense	dense
VOs in indexed trees	2	46	2	46	58
Number of Returned Data Records					
Geo-coordinates_as_Field	1	4	7	107	1004
Geohash-Grid Tree	1	4	7	107	1004
R-Tree	1	4	7	107	1004
Geohash_as_Tag	616	2774	88,864	85,459	93,404
